# Imaging Collagen Alterations in STICs and High Grade Ovarian Cancers in the Fallopian Tubes by Second Harmonic Generation Microscopy

**DOI:** 10.3390/cancers11111805

**Published:** 2019-11-16

**Authors:** Eric C. Rentchler, Kristal L. Gant, Ronny Drapkin, Manish Patankar, Paul J. Campagnola

**Affiliations:** 1 Department of Biomedical Engineering, University of Wisconsin, Madison, WI 53706, USA; rentchler@wisc.edu; 2 Department of Obstetrics and Gynecology, University of Wisconsin, Madison, WI 53706, USA; kgant@wisc.edu (K.L.G.); patankar@wisc.edu (M.P.); 3 Department of Obstetrics and Gynecology, University of Pennsylvania, Philadelphia, PA 19104, USA; drapkin@pennmedicine.upenn.edu

**Keywords:** collagen fibers, second harmonic generation, image analysis, STIC, high grade

## Abstract

The majority of high-grade serous ovarian cancers originate in the fallopian tubes, however, the corresponding structural changes in the extracellular matrix (ECM) have not been well-characterized. This information could provide new insight into the carcinogenesis and provide the basis for new diagnostic tools. We have previously used the collagen-specific Second Harmonic Generation (SHG) microscopy to probe collagen fiber alterations in high-grade serous ovarian cancer and in other ovarian tumors, and showed they could be uniquely identified by machine learning approaches. Here we couple SHG imaging of serous tubal intra-epithelial carcinomas (STICs), high-grade cancers, and normal regions of the fallopian tubes, using three distinct image analysis approaches to form a classification scheme based on the respective collagen fiber morphology. Using a linear discriminant analysis, we achieved near 100% classification accuracy between high-grade disease and the other tissues, where the STICs and normal regions were differentiated with ~75% accuracy. Importantly, the collagen in high-grade disease in both the fallopian tube and the ovary itself have a similar collagen morphology, further substantiating the metastasis between these sites. This analysis provides a new method of classification, but also quantifies the structural changes in the disease, which may provide new insight into metastasis.

## 1. Introduction

High grade serous ovarian cancer (HGSOC) is an often-fatal disease usually detected at an advanced stage when options for treatment are limited. Only ~35% of patients survive for five years when diagnosed with advanced stage disease, whereas, by comparison, ~70% of patients survive for that duration when HGSOC is detected at an early stage [1]. Unfortunately, these statistics have not changed significantly for the past several decades and there remains a clear need for more sensitive diagnostic modalities. However, standard clinical imaging modalities including CT, MRI, ultrasound, and PET do not have sufficient resolution and/or sensitivity for detection of early lesions [2,3,4]. These shortcomings are especially important for HGSOC, as small lesions can metastasize to the omentum and other locations in the peritoneum [5,6,7].

In this context, a better understanding of the carcinogenesis is also needed. Although referred to as ovarian cancer, in a majority of patients, many high-grade serous cancers originate from the secretory epithelium of the distal ends of the fallopian tube [8,9,10,11]. In this case, increased p53 expression and the presence of Serous Tubal Intraepithelial Carcinoma (STIC) lesions are two distinct precursors to HGSOC in the ovary. We hypothesize that identification of these precursors will lead to the development of more powerful diagnostic modalities for early detection of HGSOC.

Pathologists identify cancers by cellular aspects (e.g., shape, and nuclear to cytoplasmic ratio) and immunostaining of specific markers such as p53 (for ovarian cancer). However, other signatures, such as remodeling in the extracellular matrix (ECM), hold great potential in diagnosing cancers in general and HGSOC in particular, due to the early metastasis. Specifically, the collagen architecture is altered in all epithelial cancers and, for example, has been well documented in ovarian, pancreatic, and breast cancers [12,13,14,15,16,17,18,19,20,21]. The remodeling can be in the form of rearrangement of existing fibers or increased deposition of new collagen. For example, the collagen in breast cancer is characterized by perpendicularly oriented fibers to the tumor boundary [22], whereas HGSOC tumors display newly synthesized, densely packed, highly aligned wavy fibers [13,23]. Historically, collagen has been visualized by eosin in H&E staining, and more recently by picosirius red in a polarization microscope. However, these methods can only reveal the location and relative abundance of collagen, but are not highly sensitive to the collagen fiber structure.

More recently, the technique of Second Harmonic Generation (SHG) imaging microscopy has been exploited to visualize the collagen structure in several cancers (including ovarian), as well as other diseases that are characterized by alterations in collagen architecture (e.g., fibroses and connective tissue disorders) [24,25,26,27]. The SHG imaging method is performed on a laser scanning microscope (similar to two-photon excited fluorescence microscopy) and visualizes the endogenous collagen structure with great sensitivity and specificity [24,28].

Using several metrics, we have previously established that SHG imaging shows structural features of collagen architecture in the HGSOC microenvironment that are distinct from those found in other ovarian tumors and in normal ovarian stroma. For example, through detailed physical and computational analysis, we have showed that the underlying macromolecular and fibril structure in HGSOC is distinct from low-grade disease, benign tumors and normal stroma [29,30]. We have further used changes in the fiber morphology directly visualized in the SHG microscope to classify HGSOC, low-grade serous tumors, endometrioid tumors, benign tumors, high-risk tissues and normal tissues with high accuracy [13]. In this latter effort, we implemented a new form of texture analysis and machine learning. In machine learning or computer vision, texture refers to the spatial relationship of a pixel (or group of pixels) to its neighbors in the image.

We now apply, for the first time, SHG imaging and texture analysis of collagen in the normal fallopian tube, STICs and high-grade tumors. In this proof of principle study, even with limited sample numbers, we found excellent differentiation between high-grade disease and the other tissue types. Interestingly, the fibrillar collagen in the high-grade tumors in the fallopian tube has similar periodic morphology to that of HGSOC in the ovary. The observations made in this work indicate that collagen signatures can be used to detect the precursor lesions of HGSOC.

## 2. Results

### 2.1. SHG Sampling

In this work we collected two distinct SHG channels, i.e., forward and backward propagating signals. This is distinct from fluorescence, where, by widefield or confocal or multiphoton microscopy, the light emission is collected in the epi-direction. This is optimal in terms of isolating the fluorescence that forms the excitation and background rejection. However, a forward-collected fluorescence image (i.e., through the condenser) would appear with the same features as that in the epi-geometry. In contrast, due to the underlying physics, forward and backward SHG images can appear quite different. We have previously described how these components are related to fibril size and spacing and packing into fibers [31]. As a result of the underlying SHG physics, these images can contain different structural features, especially in terms of size. Thus, the information from the forward and backward SHG channels can be used as additional data for three respective analysis approaches to follow (GLCM, CT-FIRE, and 2D-FFT), as well as their combined use in a linear discriminate analysis (LDA).

**Figure 1:**
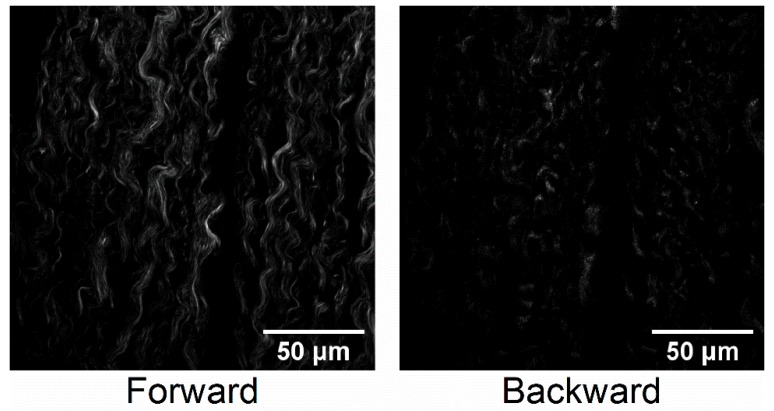
Corresponding Second Harmonic Generation (SHG) forward- and backward-collected images of a high-grade cancer in the fallopian tube, showing different image features.

Prior to image analysis, the collected stacks were split into their respective forward and backward channels. These stacks were then tested against each other to quantify the level of differences between the images, to justify their mutual inclusion in the analysis. The initial image comparison was performed in Python utilizing the scikit-image library to calculate the structural similarity index (SSIM) [32]. This index ranges from 0–1, where 1 is complete similarity in the images. These average SSIM values were distal (0.31), STIC (0.50), and HGSC (0.39), where <0.5 indicates large differences between the images. All those meeting this criterion were then included in the analysis. As an example, Figure 1 shows the respective forward and backward images of high-grade cancer in the fallopian tube, illustrating that they contain different features.

**Figure 2:**
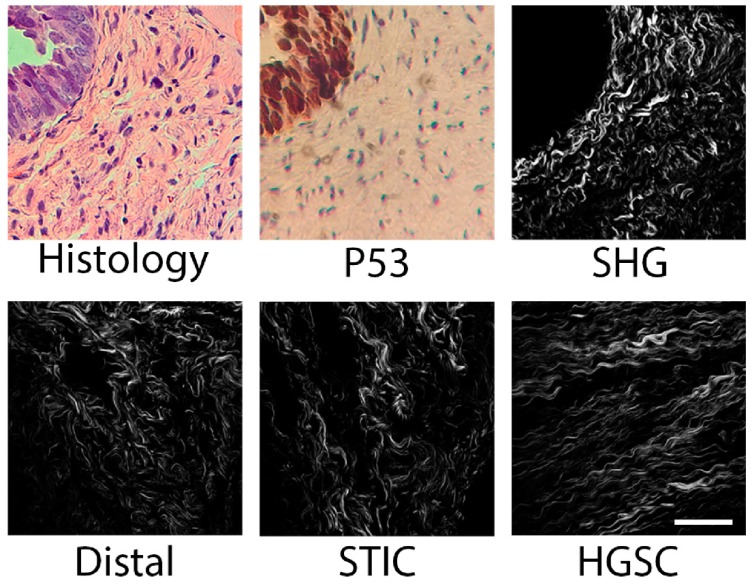
Histology and p53 stained slides were used to select regions of interest for SHG imaging. p53 stains for TP53 mutations, which are indicative of serous tubal intra-epithelial carcinomas (STIC) areas. The top row shows the H&E stain, p53 stain and SHG from the same STIC region. The bottom row shows examples of SHG images of distal, STIC, and high-grade disease in the FT used in the image analysis. Scale bar = 50 microns.

A total of 104 image stacks were utilized for the classification of the tissues and were classified into three groups: distal, STIC, and HGSOC. Visual assignment of these regions in the tissues was done prior to SHG imaging. Specifically, areas of interest were selected based on features present in the H&E and p53 stained slides, identifying dense cellular and high p53 regions. The top row of Figure 2 shows the H&E, p53, and SHG from the same STIC region. The bottom row corresponds to representative distal, STIC, and HGSOC regions in the fallopian tube used in the following analysis.

### 2.2. Gray Level Co-Occurrence Matrix Analysis

The Gray Level Co-occurrence Matrix (GLCM) is the representation of the distribution of co-occurring pixel grayscale values, i.e., it determines the number of nearest neighbors that have the same brightness. This matrix can then be used to determine several unique texture features that describe the image. We used the following features, which have well-defined formulations based on sums over adjacent pixel pairs, all neighboring pixels, or across the entire image:

(i) Angular Second Moment (ASM) and Energy measures the number of repeated pairs. The energy (square root of ASM) will be high if the occurrence of repeated pixel pairs is high.
(ii) Inverse Difference Moment (IDM) is related to the smoothness or homogeneity across the image and will be high if the gray levels of the pixel pairs are similar.
(iii) Contrast is a measure of the local contrast of an image and will be low if the gray levels of each pixel pair are similar.
(iv) Entropy measures the randomness of a gray level distribution and will be high if the gray levels are distributed randomly throughout the image.
(v) Correlation measures the linear dependency of gray levels on those of neighboring pixels.

For each region of interest imaged, the five GLCM textures were calculated for a matching forward and a backward image taken from each stack. Additionally, the images were rotated 90° and the GLCM textures were then re-calculated. This rotation was performed as the GLCM analysis works by analyzing neighboring pixels in a linear direction. Thus, if, for example, a structure in the image is oriented in the direction of analysis, the texture results will vary greatly upon 90° rotation. In contrast, randomly ordered image features will show little change in calculated texture for this rotation. The GLCM values from the 0° and 90° measurements were then averaged. By utilizing both the forward and backward images, a total of 10 textures were calculated for each region of interest imaged.

**Figure 3:**
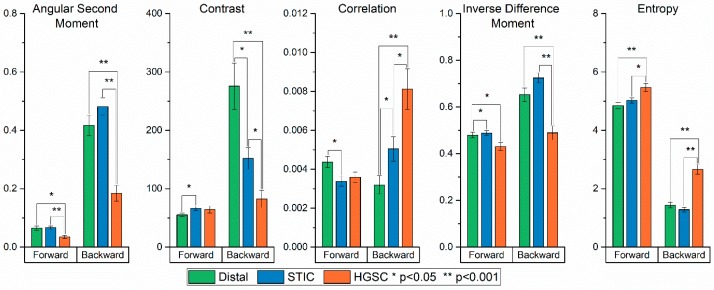
Bar plots of the mean value for each group in all five (forward and backward) of the textures calculated in the GLCM analysis. (*) and (**) correspond to *p* < 0.05 and *p* < 0.001, respectively.

The resulting values for these ten texture features of the distal, STIC and HGSOC regions with statistical differences are given in Figure 3. To determine the statistical significance between groups, a one-way ANOVA was first performed for each texture. Of the 10 textures calculated, only one (forward SHG contrast) did not show a significant difference between the groups’ means. To further corroborate the differences between groups, a t-test was performed between group pairs for each texture. Overall, the most differences were found between HGSOC, relative to the distal and STIC groups for both the forward and backward SHG images. We found the distal and STIC groups showed few differences between one another in the calculated ASM and entropy textures for both the forward-collected images. However, almost all the texture features showed significant differences between all three groups in the backward channel, suggesting increased differentiation power in this geometry.

While it is difficult to a priori determine which texture features will provide discrimination, we can understand the overall trends by considering the different contrast mechanism in these geometries. The backward collected images can reveal both smaller features as well as “segmented” fibers that result from smaller, more random fibril or fiber structures (see Figure 1), where the latter appear contiguous in the forward-detected images [31]. This phenomenon arises from destructive interference within the focal volume in the backward emitted SHG and we have previously observed these features in many tissues [33,34,35]. The distal regions and STICs show fewer of these dis-similarities than the HGSOC, which are characterized by larger numbers of these segmented features. Thus, differences in texture features based on these differences will be better revealed in the backward geometry.

As the GLCM analysis investigates pixel pairs within the image, in theory, further tuning of the analysis can be achieved by changing the distance between pixel pairs. As the initial analysis was performed utilizing only direct neighbors, testing was done with larger gaps between pixels. This, however, resulted in no significant improvement in the resulting *p*-values and that data was not included in the final classification.

### 2.3. Two-Dimensional Fast Fourier Transform Analysis

**Figure 4:**
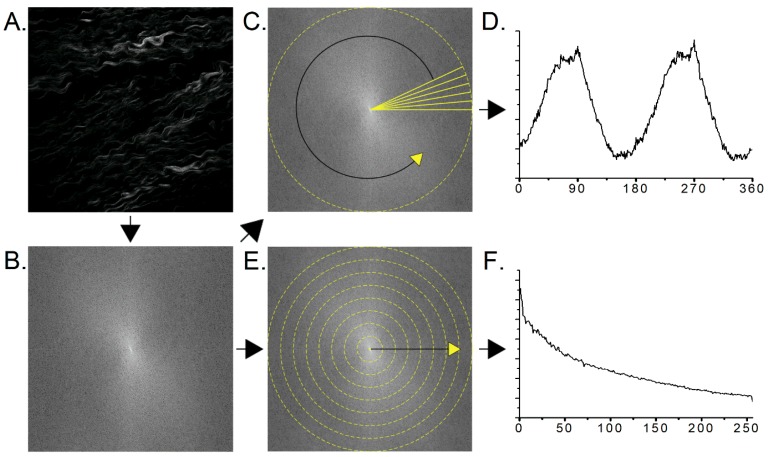
Workflow of the fast fourier transform (FFT) analysis. (**A**) starting image, (**B**) FFT of (**A**) produced in ImageJ. Two different approaches are then utilized to analyze the log-scale FFT. (**C**) Radial sums of the FFT produce the resulting intensity plot (**D**). (**E**) Circular sums of the FFT produce a curve (**F**) which is then fit with a single exponential decay. Field size = 150 × 150 µm.

We next used 2D Fast Fourier Transform (2D-FFT) analysis to compare the features present in the three tissue regions observed in both the forward and backward SHG collection channels. The FFT converts the spatial domain to that in frequency space, where, in terms of microscopy, smaller features (e.g., fibers) will have higher spatial frequencies than larger features. The specific 2D-FFT analysis here takes two different approaches to analyze the log scale power spectrum of the images produced by the native FFT function in ImageJ, where the workflow is shown in Figure 4. The first approach takes a circular selection in the center of the power spectrum and simply sums the power spectrum radially, where the resultant is then plotted as a function of angle. This plot can then be used to determine the direction of alignment in the image and/or the extent to which the image features are aligned. As the absolute directionality of the fibers within the images are not defined, we opted to use the latter approach. In this treatment, images with more organized and aligned features will produce a larger alignment value, as the resulting power spectrum becomes more asymmetrical. By contrast, an image containing random features will produce a more symmetrical power spectrum.

**Figure 5:**
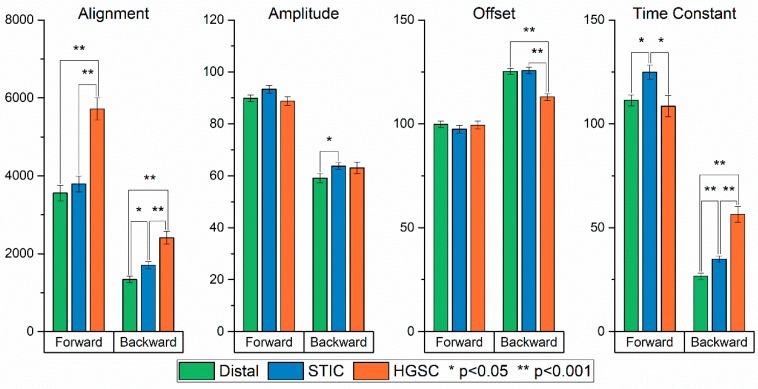
Bar plots of the mean value for each group in all four of the outputs calculated in the 2D-FFT analysis. (*) and (**) correspond to *p* < 0.05 and *p* < 0.001, respectively.

The results of the alignment measurement are shown in Figure 5. We found that the HGSOCs showed a greater alignment than the STIC and distal groups in both the forward- and backward-collected images, where the order of alignment is the same of HGSOC > STIC > distal. A one-way ANOVA was performed on the group means, and both forward and backward measurements produced *p* < 0.05 *p*-values. Between the individual groups, only the distal and STIC groups in the forward images had *p* > 0.05, showing similarity. Thus, alignment is a robust measure in differentiating the fiber architecture in these tissue classes. We note that these results are analogous to our past observations showing a greater amount of fiber alignment and organization in HGSOC compared to normal ovarian stromal tissue [36].

The second approach circularly sums the power spectrum, starting at the origin and extending out to its radius. The circular sums are then plotted as a function of the distance in pixels from the origin of the power spectrum, where this yields a single exponential decay (Figure 4F), whose amplitude, offset and time constant are the corresponding metrics. The resulting values are given in Figure 5. The amplitude in the forward and backward channels, along with the offset in the forward channel, produced no significant differences between the groups. However, the time constant in the forward and backward channels, in addition to the offset in the backward channel, showed significant differences. As the power spectrum contains information on the spatial frequencies contained in the image, the fits provide insight into the feature sizes in the respective tissues. In forward channel, the STIC group shows an increased occurrence of higher frequencies (i.e., smaller features) when compared to the distal and HGSOC groups. In the backward channel, the HGSOC group had the largest time constant, suggesting an increase in the distribution of high frequencies in the image. This increase is attributed to the punctate and segmented spots seen more prevalently in the backward images of the HGSOC group, as seen in Figure 1 [31]. Moreover, it is responsible for the differences in the forward and backward channels in the ASM and IDM in Figure 3, differentiating HGSOC from distal and STICs.

### 2.4. CT-FIRE Analysis

**Figure 6:**
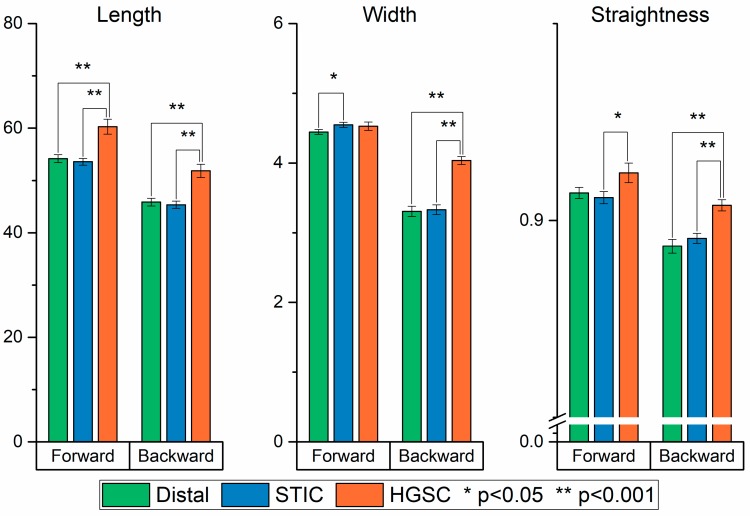
Bar plots of the mean value for each group in all three of the outputs calculated in the CT-FIRE analysis. (*) and (**) correspond to *p* < 0.05 and *p* < 0.001, respectively.

We next used curvelet transform-fiber extraction (CT-FIRE) analysis [37] to determine the length, width, and straightness of the collagen fibers in the forward and backward SHG images of the three tissues. This analysis first uses the fiber extraction (FIRE) algorithm [38] to de-noise images, enhance fiber edge features, extract fibers, followed by the curvelet transform (CT) to determine fiber metrics. The results for the forward and backward data with statistical differences are shown in Figure 6. The data reveal a high degree of similarity between the distal and STIC groups, and also highlight their dissimilarity from the HGSOC structure. For example, the results indicate that the HGSC tissues contain longer and wider fibers when compared to the distal and STIC groups (both channels). The increased width and length of the HGSOC regions correspond with our past observations of HGSOC relative to other ovarian tumors [13].

Overall, these results mirror the output of the texture features from the GLCM in Section 2.2. However, unlike those findings, there are minimal differences in the forward and backward analyses. This is because the GLCM probes neighboring features and are sensitive to the segmented features discussed above, whereas the CT-FIRE de-noises the images and is more sensitive to larger structures, such as long contiguous fibers.

### 2.5. Classification by Linear Discriminate Analysis

The three image analysis approaches had different degrees of success in delineating the three tissue types. Here, we use a linear discriminant analysis (LDA) approach combining the 24 metrics from the GCLM, 2D-FFT and CT-FIRE outputs to further classify the three tissue classes. This method combining variables can provide better discrimination than standalone metrics, where, for example, we have used it in the analysis of ovarian cancer migration data and SHG ovarian cancer imaging [30,39]. Classification is done in SAS, with the CANDISC and DISCRIM procedures classifying the tissue groups in a two-step process. CANDISC first performs a canonical discriminant analysis to derive the canonical variables for each sample that best summarizes class variation between the groups. As we have three tissue classes, two canonical variables are then calculated for each image stack. The DISCRIM procedure then utilizes the CANDISC output to develop a discriminant criterion, which is then used for group classification. Here, the data used to create the discriminant criterion is treated as an unknown and the classification accuracy is then reported. Here all the SHG forward and backward images are used, as we showed they were dis-similar (see Section 2.1) and can be treated independently.

**Figure 7:**
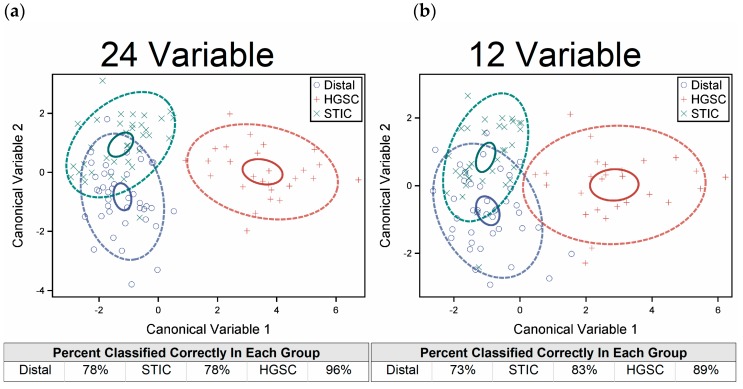
The canonical variables resulting from the CANDISC procedure using 24 (**a**) and 12 (**b**)variables. The results of the DISCRIM procedure are given in the accompanying table. Solid and dashed circles are 95% and 80% confidences, respectively. The full set of 24 metrics performed better than the reduced set of 12.

The results of the DISCRIM and CANDISC procedures utilizing all 24 of the metrics collected from the three different analysis methods are shown in Figure 7a, where the solid and dashed circles represent 95% and 80% confidence, respectively. The HGSOC group (red) is clearly distinguished from both the distal (blue) and STIC (green) groups with high classification accuracy (~96%). While the separation between the distal and STIC groups is not as good compared to HGSOC, the discriminant criterion is still able to correctly identify the distal and STIC groups with greater than 78% accuracy in both cases.

**Figure 8:**
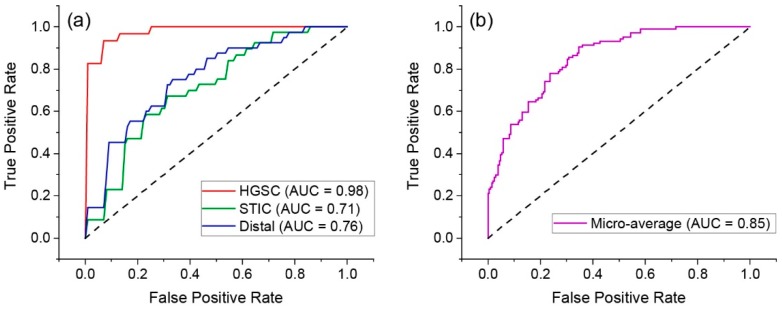
Receiver operator characteristic (ROC) curves for classification accuracy, where (**a**) and (**b**) are one vs. the rest and cohort accuracy, respectively.

We next performed receiver operator characteristic (ROC) analyses (true positive vs. false positives) using these data to uniquely quantify sensitivity and specificity. Figure 8a shows the one vs. the rest classification results, where the area under the curve yielded similar values in terms of accuracy for high-grade disease, and somewhat lower for STIC and normal regions than through the true positive accuracy analysis in Figure 7. Figure 8b shows the cohort analysis, where the area under the curve was 0.85.

As not all 24 metrics contribute equally to the discriminatory power of the combined CANDISC and DISCRIM classification, an analysis using fewer variables was done using the STEPDISC procedure. Constraining the metrics to those with a significance level less than 0.15 reduced the analysis to only 12 metrics. Running the classification with this smaller set resulted in reduced accuracy for the HGSOC to ~89%, whereas the STICs and distal regions were classified by 83% and 73%, respectively (Figure 7b). In aggregate, the full set of metrics performed better, demonstrating the value in using all the possible information.

**Figure 9:**
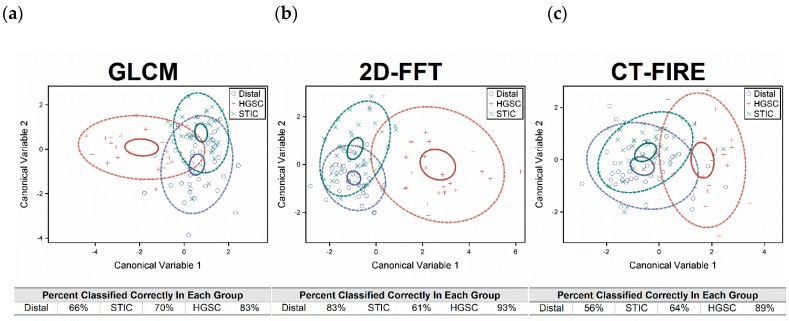
A. The CANDISC and DISCRIM results from each image analysis separately, where (**a**) is GLCM, (**b**) 2D-FFT, and (**c**) CT-FIRE results.

We next investigated the respective stand-alone ability of the GLCM, 2D-FFT and CT-FIRE analyses to discriminate the tissue groups, using the same combined CANDISC and DISCRIM classification. The results of this analysis are shown in Figure 9. Overall, the 2D-FFT metrics yielded the best classification of the three methods. Although there is a little variability, the GLCM performed better than the CT-FIRE based metrics. These findings suggest the most important differentiating fibrillar attributes. Specifically, the 2D-FFT is most sensitive to the distribution of feature sizes, as well as overall alignment. In contrast, CT-FIRE reports on the morphology of single collagen fibers, rather than the whole image pattern. The GLCM is similar in this regard, as it reveals similarity in neighboring features rather than whole fibers.

## 3. Discussion

While it is now recognized that most HGSOCs begin in the distal ends of the fallopian tubes, there have been no investigations into the associated collagen remodeling in high-grade disease or the STICs’ precursors [9,40,41,42,43,44]. The characterization has primarily been through p53 staining and H&E histology showing increased cellularity in these regions. The p53 signature serves as a marker but provides no quantitative information. Thus, quantitative examination of collagen architecture in STICs and high-grade cancer offers a significant opportunity to understand carcinogenesis as well as to provide a classification scheme. The work here builds upon our efforts, that have extensively characterized collagen alterations in a series of ovarian tumors as well as in normal stroma and those from high-risk patients [13,30]. Through systematic analysis of the collagen fiber morphology, we were able to classify high-grade regions from STIC and normal regions with almost 100% accuracy, and STICs from normal regions with high accuracy (~70%–80%). We stress that we extracted significant differences even with very small samples sets. This is because of the extensive collagen remodeling in conjunction with the sensitivity/specificity of SHG microscopy. This was also borne out in our machine learning analyses of SHG imaging of tumors in the ovary [13,36]. We note it would be interesting to have true baseline normal tissues for additional comparisons. However, due to the nature of the tumors in the FT (having high grade and STIC regions), it is also important to compare multiple collagen environments within the same TME. This has not been done previously and was a major goal of this work.

**Figure 10:**
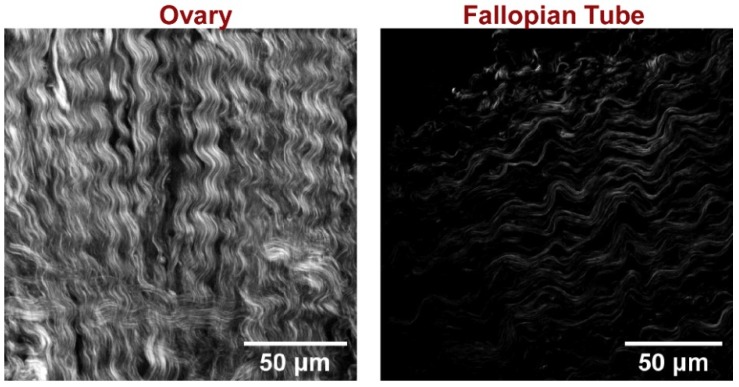
Comparison of single second harmonic generation (SHG) optical sections collected in the forward direction for HGSOC in the ovary (**left**) and fallopian tube (**right**). A similar high-frequency fiber characteristic is seen in both tissues. The SHG in the ovarian image is brighter due to denser collagen.

Characterization of the collagen architecture of HGSOC regions in the FT also provides an opportunity for direct comparison of high-grade disease in the ovary to provide further insight into its metastatic mechanism. As an example, Figure 10 shows representative SHG images of high-grade disease in these two locations. We have previously shown that the wavy pattern of the latter is highly characteristic of these tumors and is similar between patients [23,36]. The collagen in HGSOC in the fallopian tube, while less dense than that in the ovary, also has the same characteristic wavy pattern. Specifically, the fibers in both tissues have a sine wave-like morphology with similar periodicities of ~20 microns. It would be interesting to use texture analysis to show that the structures are similar. However, all of our analyses require the collagen coverage in the images to be similar, which is not the case here, and as a result the classifier found the FT and ovarian tumors to be different. We did not attempt to compare the collagen in the normal OSE and FTE, as the differences in collagen coverage were even larger. However, we note that in both normal cases the collagen is more randomly organized, with no apparent alignment.

In our previous work using classification of HGSOC images of ovarian tumors, we used an approach called “textons” [13,36]. Here, the images were broken up into pieces and convolved with a basis set of different shapes and sizes of filters, and repeating features (“textons”) were identified. The histograms of these textons were used as training sets and then compared to that of unknown images and classification was performed by ROC analyses. This scheme is a powerful approach for classification but requires significant image data. This was not limiting in our previous efforts as we had thick tissue samples available. In contrast, here we only had thin sections of the FT tissues and could not obtain sufficient data for texton utilization. Thus, we performed a more “brute force” method, involving several metrics to achieve classification. The GLCM approach yields texture features based primarily on nearest neighbor pixel intensity information across the image. The 2D-FFT evaluates spatial frequency distribution (i.e., feature sizes) as well as overall alignment, whereas the CT-FIRE extracts data on individual fiber morphology (e.g., length and width). We performed an LDA using all the combined metrics (24) and found good to excellent discrimination. The approach is fairly general and can be implemented using standard image analysis tools (ImageJ and MatLAB) and statistical analysis (e.g., SAS), even using limited data sets.

It is important to consider the translational potential of the work both in terms of potential advantages and limitations. One limitation is that cells do not provide SHG contrast. However, SHG can be readily combined with fluorescence on the same multiphoton microscope, either using dyes or cellular autofluorescence. An additional limitation of any microscope-based imaging technique is imageable sample thickness, which, depending on the tissue, is typically a few hundred microns. However, for either the fimbria or OSE, this is more than sufficient, as the most pronounced collagen changes are near the surface epithelium.

We can also consider future in vivo imaging possibilities. In theory, a laser scanning microendoscope could be constructed, either for use in conjunction with laparoscope or for insertion into the vagina and then into the fallopian tubes. Analogous devices have been proposed and designed for other applications [45,46]. These devices could utilize both two-photon excited fluorescence and backward collected SHG. Here we showed there were differences in the forward- and backward-collected SHG images in the histologic slides, where our metrics were more sensitive to the latter. Thus, this approach would be compatible with the SHG metrics in this study.

## 4. Methods

### 4.1. Fallopian Tube Tissues

This was a retrospective study where pre-existing specimens were obtained from Dr. Ronny Drapkin from a University of Pennsylvania IRB approved tissue bank. Tissues were completely de-identified, with the exception of the pathology diagnosis. Tissues were all mounted on five micron thick fixed paraffin imbedded histology slides, with some stained for p53. The specimens were from five women and the slides were either high-grade disease or both STIC and high-grade disease. There were no baseline normal samples, so distal regions to STIC and high-grade cancer were used as representatives of normal tissues.

### 4.2. SHG Microscopy

The details of the SHG microscopes in the Campagnola lab have been described in detail by Chen et al. [24] and Lien et al. [47] and are only briefly described here. Imaging is performed with laser scanning galvos (Cambridge Technologies, Bedford, MA, USA), coupled to an upright microscope (BX61; Olympus, Tokyo, Japan). The excitation source is a mode-locked Titanium Sapphire laser (Mira; Coherent, Santa Clara, CA, USA), providing 890 nm excitation. Laser scanning and data acquisition was achieved through home-written LabVIEW code and an FPGA interface board (National Instruments, Austin, Texas). The collected images were 512 × 512 pixels with a field of view of 180 × 180 µm. Power at the specimen was controlled by an electro-optic modulator (Conoptics, Danbury, CT, USA) and the average power at the focus was typically ~30 mW. Image acquisition time was 3 s per frame with three-frame Kalman averaging.

A 40 × 0.8 NA water immersion lens (LUMPlanFL/IF; Olympus, Tokyo, Japan) was used to focus the laser into the sample, and a 0.9 NA condenser collected the forward-propagating SHG signal. The lateral and axial resolution of the system was approximately 0.7 and 2.5 µm, respectively, where this is sufficient for resolving collagen fibers. The forward-directed and backward-directed SHG emission was collected using identical photon-counting detectors (7421 GaAsP; Hamamatsu, Hamamatsu City, Japan), with the backward detector in the infinity space of the microscope. For each channel, the SHG wavelength (445 nm) was isolated with a dichroic mirror and 10 nm wide bandpass filter (Semrock, Rochester, New York). The excitation wavelength was confirmed using a fiber-optic spectrometer (Ocean Optics, Dunedin, FL, USA). Circular polarization was used for imaging, as this state excites all fiber orientations equally. This polarization of the excitation laser was determined at the focus by imaging dye labelled vesicles [24].

### 4.3. Image Analysis

Gray level co-occurrence matrix (GLCM) textures were calculated using FIJI (an open-source ImageJ platform for image analysis) and a custom macro that expedited and automated the analysis. The custom macro utilized the Texture Analyzer plugin (Julio E. Cabrera, version v0.4 2006/07/07) to calculate five texture parameters: Angular Second Moment (ASM), Inverse Difference Moment (IDM), Contrast, Entropy, and Correlation. FIJI was also utilized in the two-dimension fast Fourier transform (2D-FFT) analysis, where two custom macros utilized the Radial Profile Extended (Philippe Carl, version 2017/04/18) and the Oval Profile Plot (Bill O’Connell, version 2012/03/01) plugins. All curve fitting of the 2D-FFT data was done in Origin 2016 (OriginLab, Northampton, MA, USA). CT-FIRE was utilized to perform the curvelet transform and fiber extraction to characterize individual fiber morphology features [37].

### 4.4. Statistical Analysis

Canonical discriminant analysis and discriminant criterion classification were performed in SAS (SAS Institute Inc., Chicago, IL, USA) using the CANDISC and DISCRIM procedures. The data included in classification were selected through a stepwise discriminant analysis that is also performed in SAS using the STEPDISC procedure. All ANOVA statistical tests and curve fitting were performed in Origin 2016. *p* values of <0.05 were considered significant.

## 5. Conclusions

Using SHG microscopy we have performed a detailed analysis of the fiber morphology in STICs and high-grade cancers in the fallopian tubes relative to normal tissues. We found excellent classification between HGSOC and the other regions and good differentiation between STICs and normal regions. Interestingly, the wavy fiber morphology seen in high-grade disease is similar (based on periodicity) between the fallopian tube and the ovary itself.

The work has both short- and long-term clinical significance. The analysis of the collagen architecture could be incorporated into pathology practice. The SHG microscope for this purpose could essentially be an automated slide reader with an appropriate laser. While we used a laser with a large footprint, much smaller lasers are commercially available that would work for this purpose. Additionally, the collagen in fallopian tubes removed during salpingectomy could be analyzed and used for risk prediction of ovarian cancer. This could potentially preserve the ovary for some women. In the long term, an SHG endoscopic probe could be developed to image the collagen structure in the fimbria. This is a difficult task, given tortuosity the nature of the fallopian tubes, but it may be solved in the future, as the development of in vivo SHG probes is an active area of research.

## References

[1] Torre L.A., Trabert B., DeSantis C.E., Miller K.D., Samimi G., Runowicz C.D., Gaudet M.M., Jemal A., Siegel R.L. (2018). Ovarian cancer statistics, 2018. CA Cancer J. Clin..

[2] Karlan B.Y. (1997). The status of ultrasound and color Doppler imaging for the early detection of ovarian carcinoma. Cancer Investig..

[3] Qayyum A., Coakley F.V., Westphalen A.C., Hricak H., Okuno W.T., Powell B. (2005). Role of CT and MR imaging in predicting optimal cytoreduction of newly diagnosed primary epithelial ovarian cancer. Gynecol. Oncol..

[4] Bristow R.E., Giuntoli R.L., Pannu H.K., Schulick R.D., Fishman E.K., Wahl R.L. (2005). Combined PET/CT for detecting recurrent ovarian cancer limited to retroperitoneal lymph nodes. Gynecol. Oncol..

[5] Doubeni C.A., Doubeni A.R., Myers A.E. (2016). Diagnosis and Management of Ovarian Cancer. Am Fam. Physician.

[6] van Nagell J.R., DePriest P.D., Reedy M.B., Gallion H.H., Ueland F.R., Pavlik E.J., Kryscio R.J. (2000). The efficacy of transvaginal sonographic screening in asymptomatic women at risk for ovarian cancer. Gynecol. Oncol..

[7] Fishman D.A., Cohen L., Blank S.V., Shulman L., Singh D., Bozorgi K., Tamura R., Timor-Tritsch I., Schwartz P.E. (2005). The role of ultrasound evaluation in the detection of early-stage epithelial ovarian cancer. Am. J. Obs. Gynecol..

[8] Crum C.P., Drapkin R., Miron A., Ince T.A., Muto M., Kindelberger D.W., Lee Y. (2007). The distal fallopian tube: A new model for pelvic serous carcinogenesis. Curr. Opin. Obs. Gynecol..

[9] Folkins A.K., Jarboe E.A., Saleemuddin A., Lee Y., Callahan M.J., Drapkin R., Garber J.E., Muto M.G., Tworoger S., Crum C.P. (2008). A candidate precursor to pelvic serous cancer (p53 signature) and its prevalence in ovaries and fallopian tubes from women with BRCA mutations. Gynecol. Oncol..

[10] Labidi-Galy S.I., Papp E., Hallberg D., Niknafs N., Adleff V., Noe M., Bhattacharya R., Novak M., Jones S., Phallen J. (2017). High grade serous ovarian carcinomas originate in the fallopian tube. Nat. Commun..

[11] Mehra K., Mehrad M., Ning G., Drapkin R., McKeon F.D., Xian W., Crum C.P. (2011). STICS, SCOUTs and p53 signatures; a new language for pelvic serous carcinogenesis. Front Biosci. (Elite Ed).

[12] Ricciardelli C., Rodgers R.J. (2006). Extracellular matrix of ovarian tumors. Semin. Reprod. Med..

[13] Wen B., Campbell K.R., Tilbury K., Nadiarnykh O., Brewer M.A., Patankar M., Singh V., Eliceiri K.W., Campagnola P.J. (2016). 3D texture analysis for classification of second harmonic generation images of human ovarian cancer. Sci. Rep.-UK.

[14] Cicchi R., Kapsokalyvas D., De Giorgi V., Maio V., Van Wiechen A., Massi D., Lotti T., Pavone F.S. (2010). Scoring of collagen organization in healthy and diseased human dermis by multiphoton microscopy. J. Biophotonics.

[15] Drifka C.R., Eliceiri K.W., Weber S.M., Kao W.J. (2013). A bioengineered heterotypic stroma-cancer microenvironment model to study pancreatic ductal adenocarcinoma. Lab Chip.

[16] Drifka C.R., Loeffler A.G., Mathewson K., Keikhosravi A., Eickhoff J.C., Liu Y., Weber S.M., Kao W.J., Eliceiri K.W. (2016). Highly aligned stromal collagen is a negative prognostic factor following pancreatic ductal adenocarcinoma resection. Oncotarget.

[17] Conklin M.W., Eickhoff J.C., Riching K.M., Pehlke C.A., Eliceiri K.W., Provenzano P.P., Friedl A., Keely P.J. (2011). Aligned collagen is a prognostic signature for survival in human breast carcinoma. Am J. Pathol..

[18] Rueden C.T., Conklin M.W., Provenzano P.P., Keely P.J., Eliceiri K.W. (2009). Nonlinear optical microscopy and computational analysis of intrinsic signatures in breast cancer. Conf. Proc. IEEE Eng. Med. Biol. Soc..

[19] Provenzano P.P., Inman D.R., Eliceiri K.W., Keely P.J. (2009). Matrix density-induced mechanoregulation of breast cell phenotype, signaling and gene expression through a FAK-ERK linkage. Oncogene.

[20] Provenzano P.P., Inman D.R., Eliceiri K.W., Knittel J.G., Yan L., Rueden C.T., White J.G., Keely P.J. (2008). Collagen density promotes mammary tumor initiation and progression. BMC Med..

[21] Brown E., McKee T., diTomaso E., Pluen A., Seed B., Boucher Y., Jain R.K. (2003). Dynamic imaging of collagen and its modulation in tumors in vivo using second-harmonic generation. Nat. Med..

[22] Provenzano P.P., Eliceiri K.W., Campbell J.M., Inman D.R., White J.G., Keely P.J. (2006). Collagen reorganization at the tumor-stromal interface facilitates local invasion. BMC Med..

[23] Nadiarnykh O., Lacomb R.B., Brewer M.A., Campagnola P.J. (2010). Alterations of the extracellular matrix in ovarian cancer studied by Second Harmonic Generation imaging microscopy. BMC Cancer.

[24] Chen X., Nadiarynkh O., Plotnikov S., Campagnola P.J. (2012). Second harmonic generation microscopy for quantitative analysis of collagen fibrillar structure. Nat. Protoc..

[25] Campagnola P.J., Dong C.Y. (2011). Second harmonic generation microscopy: Principles and applications to disease diagnosis. Lasers Photonics Rev..

[26] Pena A.M., Fabre A., Debarre D., Marchal-Somme J., Crestani B., Martin J.L., Beaurepaire E., Schanne-Klein M.C. (2007). Three-dimensional investigation and scoring of extracellular matrix remodeling during lung fibrosis using multiphoton microscopy. Microsc. Res. Tech..

[27] Sun W., Chang S., Tai D.C., Tan N., Xiao G., Tang H., Yu H. (2008). Nonlinear optical microscopy: Use of second harmonic generation and two-photon microscopy for automated quantitative liver fibrosis studies. J. Biomed. Opt..

[28] Campagnola P.J., Loew L.M. (2003). Second-harmonic imaging microscopy for visualizing biomolecular arrays in cells, tissues and organisms. Nat. Biotechnol..

[29] Campbell K.R., Chaudhary R., Handel J.M., Patankar M.S., Campagnola P.J. (2018). Polarization-resolved second harmonic generation imaging of human ovarian cancer. J. Biomed. Opt..

[30] Tilbury K.B., Campbell K.R., Eliceiri K.W., Salih S.M., Patankar M., Campagnola P.J. (2017). Stromal alterations in ovarian cancers via wavelength dependent Second Harmonic Generation microscopy and optical scattering. BMC Cancer.

[31] Lacomb R., Nadiarnykh O., Townsend S.S., Campagnola P.J. (2008). Phase Matching considerations in Second Harmonic Generation from tissues: Effects on emission directionality, conversion efficiency and observed morphology. Opt. Commun..

[32] Wang Z., Bovik A.C., Sheikh H.R., Simoncelli E.P. (2004). Image quality assessment: From error visibility to structural similarity. IEEE T Image Process.

[33] Nadiarnykh O., LaComb R.B., Campagnola P.J., Mohler W.A. (2007). Coherent and incoherent SHG in fibrillar cellulose matrices. Opt. Express.

[34] Nadiarnykh O., Plotnikov S., Mohler W.A., Kalajzic I., Redford-Badwal D., Campagnola P.J. (2007). Second Harmonic Generation imaging microscopy studies of Osteogenesis Imperfecta. J. Biomed. Opt..

[35] Campbell K.R., Chaudhary R., Montano M., Iozzo R.V., Bushman W.A., Campagnola P.J. (2019). Second-harmonic generation microscopy analysis reveals proteoglycan decorin is necessary for proper collagen organization in prostate. J. Biomed. Opt..

[36] Wen B.L., Brewer M.A., Nadiarnykh O., Hocker J., Singh V., Mackie T.R., Campagnola P.J. (2014). Texture analysis applied to second harmonic generation image data for ovarian cancer classification. J. Biomed. Opt..

[37] Bredfeldt J.S., Liu Y., Pehlke C.A., Conklin M.W., Szulczewski J.M., Inman D.R., Keely P.J., Nowak R.D., Mackie T.R., Eliceiri K.W. (2014). Computational segmentation of collagen fibers from second-harmonic generation images of breast cancer. J. Biomed. Opt..

[38] Stein A.M., Vader D.A., Jawerth L.M., Weitz D.A., Sander L.M. (2008). An algorithm for extracting the network geometry of three-dimensional collagen gels. J. Microsc..

[39] Ajeti V., Lara-Santiago J., Alkmin S., Campagnola P.J. (2017). Ovarian and Breast Cancer Migration Dynamics on Laminin and Fibronectin Bidirectional Gradient Fibers Fabricated via Multiphoton Excited Photochemistry. Cell Mol. Bioeng..

[40] Kuhn E., Kurman R.J., Vang R., Sehdev A.S., Han G., Soslow R., Wang T.L., Shih Ie M. (2012). TP53 mutations in serous tubal intraepithelial carcinoma and concurrent pelvic high-grade serous carcinoma--evidence supporting the clonal relationship of the two lesions. J. Pathol..

[41] Marquez R.T., Baggerly K.A., Patterson A.P., Liu J., Broaddus R., Frumovitz M., Atkinson E.N., Smith D.I., Hartmann L., Fishman D. (2005). Patterns of gene expression in different histotypes of epithelial ovarian cancer correlate with those in normal fallopian tube, endometrium, and colon. Clin. Cancer Res..

[42] Vang R., Shih Ie M., Kurman R.J. (2009). Ovarian low-grade and high-grade serous carcinoma: Pathogenesis, clinicopathologic and molecular biologic features, and diagnostic problems. Adv. Anta Pathol..

[43] Piek J.M., van Diest P.J., Verheijen R.H. (2008). Ovarian carcinogenesis: An alternative hypothesis. Adv. Exp. Med. Biol..

[44] Vang R., Shih Ie M., Kurman R.J. (2013). Fallopian tube precursors of ovarian low- and high-grade serous neoplasms. Histopathology.

[45] Wu Y.C., Leng Y.X., Xi J.F., Li X.D. (2009). Scanning all-fiber-optic endomicroscopy system for 3D nonlinear optical imaging of biological tissues. Opt. Express.

[46] Sanchez G.N., Sinha S., Liske H., Chen X., Nguyen V., Delp S.L., Schnitzer M.J. (2015). In Vivo Imaging of Human Sarcomere Twitch Dynamics in Individual Motor Units. Neuron.

[47] Lien C.H., Tilbury K., Chen S.J., Campagnola P.J. (2013). Precise, motion-free polarization control in Second Harmonic Generation microscopy using a liquid crystal modulator in the infinity space. Biomed. Opt. Express.

